# Automatic vocalisation-based detection of fragile X syndrome and Rett syndrome

**DOI:** 10.1038/s41598-022-17203-1

**Published:** 2022-08-03

**Authors:** Florian B. Pokorny, Maximilian Schmitt, Mathias Egger, Katrin D. Bartl-Pokorny, Dajie Zhang, Björn W. Schuller, Peter B. Marschik

**Affiliations:** 1 iDN – interdisciplinary Developmental Neuroscience, Division of Phoniatrics, Medical University of Graz, Graz, Austria; 2 Machine Intelligence & Signal Processing group (MISP), Technical University of Munich, Munich, Germany; 3 EIHW – Chair of Embedded Intelligence for Health Care and Wellbeing, University of Augsburg, Augsburg, Germany; 4 GLAM – Group on Language, Audio, & Music, Imperial College London, London, UK; 5 Department of Child and Adolescent Psychiatry and Psychotherapy, University Medical Center Göttingen, Göttingen, Germany; 6 Leibniz ScienceCampus Primate Cognition, Göttingen, Germany; 7 Center of Neurodevelopmental Disorders (KIND), Center for Psychiatry Research, Department of Women’s and Children’s Health, Karolinska Institutet, Stockholm, Sweden

**Keywords:** Neuroscience, Medical research, Engineering

## Abstract

Fragile X syndrome (FXS) and Rett syndrome (RTT) are developmental disorders currently not diagnosed before toddlerhood. Even though speech-language deficits are among the key symptoms of both conditions, little is known about infant vocalisation acoustics for an automatic earlier identification of affected individuals. To bridge this gap, we applied intelligent audio analysis methodology to a compact dataset of 4454 home-recorded vocalisations of 3 individuals with FXS and 3 individuals with RTT aged 6 to 11 months, as well as 6 age- and gender-matched typically developing controls (TD). On the basis of a standardised set of 88 acoustic features, we trained linear kernel support vector machines to evaluate the feasibility of automatic classification of (a) FXS vs TD, (b) RTT vs TD, (c) atypical development (FXS+RTT) vs TD, and (d) FXS vs RTT vs TD. In paradigms (a)–(c), all infants were correctly classified; in paradigm (d), 9 of 12 were so. Spectral/cepstral and energy-related features were most relevant for classification across all paradigms. Despite the small sample size, this study reveals new insights into early vocalisation characteristics in FXS and RTT, and provides technical underpinnings for a future earlier identification of affected individuals, enabling earlier intervention and family counselling.

## Introduction

Infant development refers to structural and functional changes in the human organism within the first year of postnatal life. These changes are reflected in different developmental domains, such as the cognitive domain, the motor domain, or the speech-language domain. For instance, the latter reveals a sequence of vocal transformations characterising the transition from protophones predominant within the first weeks of life to first meaningful words typically produced at around one year of age^1–8^. However, sometimes developmental processes appear deviant from what is considered to be within the normal range. For example, this can become manifest in behavioural peculiarities and/or in a delayed achievement or even the non-achievement of developmental milestones. A developmental disorder is a medical condition characterised by deficits in at least one developmental domain^9^. Most developmental disorders become manifest very early in life^9^. Some are already apparent at birth or even earlier, such as trisomy 21 (Down syndrome), due to specific physical and/or behavioural features. In contrast, there are developmental disorders that lack obvious early signs. In these disorders, early development seems to proceed apparently inconspicuously until a certain threshold of deviance from neurotypical development is reached and a clinical diagnosis can be made. In some disorders, this does not happen before toddlerhood^10^. A well-known ‘late detected’ developmental disorder is the autism spectrum disorder (ASD). Even though a number of candidate genes and environmental factors have been increasingly discussed in connection with ASD, its exact etiology remains unclear so far^9,11–14^. Some other late detected developmental disorders have a confirmed genetic cause, such as the rare conditions fragile X syndrome (FXS) or Rett syndrome (RTT).

Currently known as the most prevalent heritable cause of intellectual disability^15^, FXS was first described in 1943 as a ‘mental defect’ by the British geneticists James Purdon Martin and Julia Bell^16^. The syndrome owes its name to a mutation in the X chromosome-linked Fragile X Mental Retardation 1 (*FMR1*) gene being responsible for the condition^17,18^. FXS has a prevalence of about 1 in 7000 males and 1 in 11,000 females^19^. Its manifestation is usually milder in affected females than in males^20^ leading to a higher mean age of diagnosis for females—around 42 months—compared to 36 months for males^21^.

RTT is a profound neurological disorder. It was named after the Austrian neuropaediatrician Andreas Rett, who was the first to document a group of affected patients in 1966^22,23^. Thirty-three years later, de novo mutations in the X chromosome-linked gene encoding Methyl-CpG-binding Protein 2 (*MECP2*) were identified to be the main cause of RTT^24^. RTT occurs in about 1 of 10,000 live female births^25^; most affected males decease in the prenatal period^26^, though an increasing number of surviving males with an *MECP2* mutation is being reported ^27^. Among these are, for instance, males with Klinefelter syndrome (i.e., they have one Y chromosome and two X chromosomes), who have one X chromosome with a mutated *MECP2* gene, as well as males, who have mosaic *MECP2* mutations (i.e., cells containing the *MECP2* mutation exist alongside a normal cell line). On average, individuals with RTT are diagnosed at around 32 months^28^ on the basis of clinical consensus criteria targeting on specific motor abnormalities and atypicalities in the use of expressive language^29^.

Deficits in the speech-language domain are a core characteristic of both RTT^29,30^ and FXS^20,31,32^. Nevertheless, there is limited knowledge of early verbal capacities of individuals with FXS or RTT, i.e., of verbal behaviour from the prodromal period^33–36^. In a study comparing the (pre-)linguistic development of 10 individuals with FXS and 14 typically developing (TD) individuals at 9–12 months of age, Belardi and colleagues^37^ reported a reduced number of produced syllables per time and a reduced proportion of produced canonical syllables for the individuals with FXS. Preliminary results by Hamrick and colleagues^38^ on a group of 22 individuals with FXS and a group of 17 TD controls indicate that, associations between selected characteristics of verbal behaviour at 9 months, such as the mean fundamental frequency ($\textit{f}_{o}$) or $\textit{f}_{o}$ range, and language outcomes at 24 months might differ between the groups. In a longitudinal study on 15 individuals with RTT over the first 2 years of life by Marschik and colleagues^39^, some individuals were found not to acquire certain (pre-)linguistic capacities, such as the reduplication of canonical syllables or the production of (proto-)words. Only 1 of 15 individuals was observed to produce word combinations. Moreover, atypical vocal patterns, such as phases of high-pitched crying-like, ingressive, or pressed phonation, were repeatedly reported in connection with early verbal behaviour of individuals with RTT^39–42^. Such patterns were found to occur in alteration with apparently typical vocal patterns^43^. However, although these previous studies could raise the evidence of early verbal peculiarities in individuals with FXS or RTT, detailed acoustic investigations of early audio-recorded FXS- and RTT-related verbal behaviour at the signal level are still pending.

The aim of the present study was to bridge this gap by keeping up with the rising age of intelligent audio analysis. Intelligent audio analysis can be regarded as a combination of advanced audio signal processing and machine learning^44^. Building the methodological basis for a wide range of applications—all in the area of automatic analysis and classification of recorded audio signals—intelligent audio analysis has significantly influenced the field of computational paralinguistics^45^ and the related ongoing series of annual (Interspeech) Computational Paralinguistics Challenges^46,47^. Typical computational paralinguistics tasks are the automatic categorisation of recorded speech or the audio-based retrieval of speech-related meta-information, such as age and gender^48^, emotional state^46,49–51^, or the medical condition of the speaker^44,47,52,53^. Over the last years, computational paralinguistics has sporadically also focused on the classification of infant verbal behaviour, such as the automatic differentiation of mood-related^50^ or pre-linguistic^54^ types of infant sounds.

In 2016, we ran the very first experiments on an automatic early verbal behaviour-based recognition of RTT vs typical development (TD) and achieved a promising unweighted average recall of 76.5 %^55^. With the present study, we intended to continue with what we have initiated by expanding a methodologically improved intelligent infant vocalisation analysis approach for another late detected genetic condition—FXS. On the basis of a compact dataset of recorded verbal behaviour of individuals with FXS, RTT, and TD aged 6 to 11 months, different analysis scenarios were addressed. This includes scenarios in which the data of individuals with FXS, individuals with RTT, and TD individuals were treated in one and the same model. By focusing on the following two research questions, we aimed to provide technical underpinnings for an early verbal behaviour-based detection of atypical development (AD) using the example of FXS and RTT: (1) Can the early verbal behaviour of individuals with FXS, individuals with RTT, and TD individuals be automatically differentiated based on acoustic characteristics? (2) Which acoustic characteristics contribute most in the attempt to automatically differentiate between the early verbal behaviour of individuals with FXS, individuals with RTT, and TD individuals? To the best of our knowledge, this is the first intelligent infant vocalisation analysis study on FXS and the first multi-class approach on data of two late detected genetic disorders. Our work shall lay the cornerstone for a reliable verbal behaviour-based earlier detection of currently late detected developmental disorders such as FXS and RTT in the future, facilitating an earlier entry into intervention for affected individuals as well as earlier counselling of their families.

## Methods

In this study, we combine acoustic signal processing and machine learning methodology in a retrospective data analysis approach. In particular, data of infant verbal behaviour were retrospectively collected^56^ and analysed at a time each participant’s developmental outcome had already been known. The study was approved by the Institutional Review Board of the Medical University of Graz, Austria (27-388 ex 14/15) and all experiments were performed in accordance with the relevant guidelines and regulations.

### Participants

The data of twelve participants were taken from the research database of the Research Unit iDN—interdisciplinary Developmental Neuroscience, at the Medical University of Graz, Austria, i.e., the Graz University Audiovisual Research Database for the Interdisciplinary Analysis of Neurodevelopment (GUARDIAN)^57,58^. Inclusion criteria were that all participants were born at term age from singleton pregnancies and grew up in German-speaking families. Six participants developed typically (TD group; TD1–TD6), of which three were males (TD♂ group; TD1–TD3) and three were females (TD♀ group; TD4–TD6). The remaining six participants developed atypically (AD group), i.e., they were diagnosed with a developmental disorder. Three participants of the AD group were males and received a diagnosis of FXS (FXS group; FXS1–FXS3), three were females and received a diagnosis of typical RTT (RTT group; RTT1–RTT3). Genetic testing in the participants with RTT revealed the following pathogenic *MECP2* mutations: p.R106W for RTT1, p.F157L for RTT2, and p.R168X for RTT3. Diagnoses were not made before toddlerhood. In the participants FXS1–FXS3, the exact age of diagnosis was 13 mo, 18 mo, and 45 mo, respectively.

### Material

The study dataset comprised 17 h and 50 mins of audio-video clips showing the participants in their second half year of life. For each group, material from the 7th until the 12th month of age was available. Recordings were made by the participants’ parents to document family life in typical home settings, such as in playing, bathing, or feeding situations. At the time of recording, parents of participants with FXS or RTT were not aware of their children’s medical condition, yet. The material was provided by the participants’ parents (FXS group, RTT group) or the participants themselves (TD group). Informed consent was obtained from all participants and/or their legal guardian(s).

### Segmentation

The entire audio-video material was screened and manually segmented for infant verbal behaviour by a research assistant (M.E.) using the coding tool The Observer XT by Noldus (https://www.noldus.com/observer-xt [as of 11 October 2021]). Considering respiratory activity as the underlying process of voice production, segmentation was done following the strategy to assign each verbal behaviour to a distinct vocal breathing group^59,60^. According to this, start and stop of each segment had to coincide with a phase of inhalation. Ingressive sounds were defined not to mark segment boundaries, but to be treated as regular (parts of) segments. Isolated vegetative sounds, such as coughing sounds, burping sounds, or hiccups were not segmented. The segmentation output was rechecked by the first author (F.B.P.). Verified segments were exported as audio clips in the format 44.1 kHz/16 bits/single-channel/PCM for further processing. Henceforth, these segments are referred to as vocalisations. The segmentation process resulted in a total of 4644 vocalisations.

Next, two independent listeners experienced in infant sound classification (K.D.B. and a PhD student in experimental psychology) annotated the corpus for infant mood. For each vocalisation, they rated whether it had been produced in either neutral or non-neutral mood. The latter category included both positive-mood vocalisations, i.e., laughing or pleasure bursts, and negative-mood vocalisations, i.e., distress patterns, such as crying or fussing. Annotations were accepted if both listeners voted for the same category. In case of disagreement, a third independent listener (F.B.P.) was consulted to obtain a majority decision. During the mood annotation process, all three raters were blind for participant group assignments. One hundred ninety vocalisations were identified as non-neutral-mood vocalisations. Generally, both positive-mood and negative-mood vocalisations are characterised by specific acoustic patterns, e.g., with regard to pitch and loudness, distinct from patterns in neutral-mood vocalisations. Due to the unbalanced distribution of these acoustically prominent vocalisations across the dataset, we decided to exclude them from this study to ensure inter-group comparability. Thus, the final study dataset consisted of 4454 pre-linguistic vocalisations produced in neutral mood.

The participant-wise and group-wise numbers of included vocalisations per duration of available audio(-video) material are given in Table 1. For all participant groups, vocalisations from each month of the second half year of life were available. The mean duration of included vocalisations was 1.79 s with a standard deviation of 1.37 s. The median vocalisation duration was 1.39 s. The shortest vocalisation in the dataset was an isolated vowel-like sound^7^ by participant TD1 at 6 months of age. It had a duration of 232 ms. The longest vocalisation was an extended canonical babbling sequence^7^ by participant TD4 at 8 months of age and had a duration of 16.86 s.

**Table 1:**
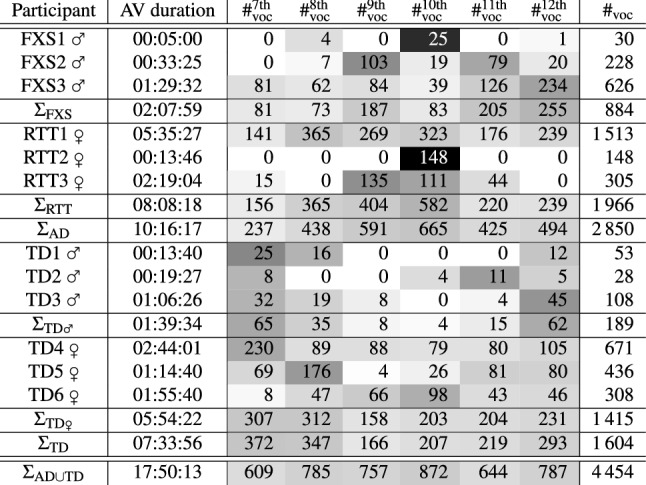
Available audio-video (AV) duration in format hh:mm:ss (two-digit hour number:two-digit minute number:two-digit second number; seconds rounded down to integer values; sums calculated on the basis of exact durations) as well as month-wise (${\#}^{\text {7th, 8th, 9th, 10th, 11th, 12th}}_{\text {voc}}$) and overall ($\#_{voc}$) number of included vocalisations per participant, per group, and in total.

AD, atypical development; FXS, fragile X syndrome; RTT, Rett syndrome; TD, typical development; ♀, female; ♂, male; colour code for month-wise numbers of vocalisations: greyscale proportional to the total number of vocalisations per line between white = 0 % of vocalisations and black = 100 % of vocalisations.

### Analysis

To answer the posed research questions (see “Introduction”), analysis in this study was twofold. On the one hand, we evaluated the feasibility of automatic vocalisation classification, i.e., the automatic vocalisation-based identification of individuals with FXS, individuals with RTT, and TD individuals (see “Classification”). On the other hand, we tried to identify those early acoustic vocalisation characteristics that bear most information to automatically differentiate between individuals with FXS, individuals with RTT, and TD individuals (see “Feature analysis”). Throughout this study, we followed a gender-matched experimental design. Four paradigms were addressed: (a) FXS group vs TD♂ group (3♂ vs 3♂ participants); (b) RTT group vs TD♀ group (3♀ vs 3♀ participants); (c) AD group (= FXS group + RTT group) vs TD group (= TD♂ group + TD♀ group) (3♂ + 3♀ vs 3♂ + 3♀ participants); (d) FXS group vs RTT group vs TD group (3♂ vs 3♀ vs 3♂ + 3♀ participants). We decided not to treat the exclusive paradigm FXS group vs RTT group on the basis of this dataset, as it would have coincided with a male vs female participants differentiation paradigm.

#### Feature extraction

Automatic vocalisation classification and vocalisation characteristics analysis were based on acoustic features, i.e., mathematical descriptors used to acoustically characterise each vocalisation in an informative, but ideally non-redundant way^44,56^. To ensure reproducibility, feature extraction was done by means of the widely used open-source tool kit openSMILE^61,62^ by audEERING GmbH (https://audeering.com/technology/opensmile [as of 11 October 2021]). Representing the state of the art in compactness at high performance, we decided to apply the extended Geneva Minimalistic Acoustic Parameter Set (eGeMAPS^63^). The eGeMAPS is the most current standardised feature set of openSMILE. It was compiled by voice and speech scientists based on theoretical and empirical criteria to allow for efficient baseline evaluations in the area of automatic voice and speech analysis. Extracting the eGeMAPS features implicates a defined two-level calculation process. On the first level, a broad range of acoustic frequency-related, spectral/cepstral, and energy/amplitude-related descriptors, such as the $\textit{f}_{o}$, Mel-frequency cepstral coefficients (MFCCs), and loudness are extracted on a short-term basis. Specifically, first-level descriptors are calculated for overlapping time windows of either 20 ms (e.g., loudness) or 60 ms (e.g., $\textit{f}_{o}$) alongside the input audio signal at a step size of 10 ms. On the second level, selected statistical functionals, such as the arithmetic mean and the standard deviation, are applied to the trajectories of the first-level descriptors. Thereby, second-level descriptors, such as the mean $\textit{f}_{o}$ of the input signal, are generated. Generally, second-level descriptors are calculated for all regions of the first-level descriptor trajectories. Some second-level descriptors are (additionally) calculated explicitly for specific temporal-related regions of the first-level descriptor trajectories, such as for voiced regions ($\textit{f}_{o}$ existent), unvoiced regions ($\textit{f}_{o}$ non-existent), rising contour slopes, and falling contour slopes. Moreover, there are second-level descriptors that exclusively build upon temporal-related vocalisation information, e.g., the mean length of continuous voiced regions. The eGeMAPS comprises 88 defined second-level descriptors, of which 24 represent the group of frequency-related features, 43 the group of spectral/cepstral (balance/shape/dynamics) features, 15 the group of energy/amplitude-related features, and 6 the group of temporal features. In this study, second-level feature processing was applied to the respective full length of each vocalisation. Thus, each vocalisation was finally represented by a single feature vector of dimension 88. This was the input representation for classification.

#### Classification

We applied machine learning methodology to automatically classify vocalisations according to participant groups, henceforth also referred to as classes. We decided to employ linear kernel support vector machines (SVMs) as classifier due to their widespread use for baseline evaluations in computational paralinguistics tasks over the last years^49,50,52–54,64,65^, including tasks on small- to moderate-sized datasets. For the two-class paradigms (a)–(c) (see “Analysis”), we followed a leave-one-speaker-pair-out (LOSpO) cross-validation procedure. In a first step, participants of different groups were paired to each other according to their respective number of available vocalisations in the dataset. For example, in paradigm (a) FXS vs TD♂, the participant with FXS with the highest number of vocalisations (FXS3) was paired with the male TD participant with the highest number of vocalisations (TD3), the participant with FXS with the second highest number of vocalisations (FXS2) was paired with the male TD participant with the second highest number of vocalisations (TD1), and finally, the participant with FXS with the lowest number of vocalisations (FXS1) was paired with the male TD participant with the lowest number of vocalisations (TD2). Thus, both for paradigm (a) FXS vs TD♂ and (b) RTT vs TD♀, three speaker pairs were defined, respectively. For paradigm (c) AD vs TD, the pairs for paradigms (a) and (b) were maintained resulting in six gender-matched pairs. For the three-class paradigm (d) FXS vs RTT vs TD, three participant quadruples of each one participant with FXS, one participant with RTT, and one male and one female TD participant were defined, again considering the participant-wise numbers of available vocalisations. This was equivalent to pooling together the same-ranked pairs for paradigms (a) and (b), respectively. In each iteration of the cross-validation procedure, the classifier was trained on the basis of vocalisations of all participant pairs/quadruples except for one (training partition), and subsequently, optimised and tested on vocalisations of the remaining participant pair/quadruple (test partition). Each participant pair/quadruple was used as test partition exactly one time throughout the cross-validation procedure. Thus, for paradigms (a), (b), and (d), three iterations were executed each, for paradigm (c), six iterations.

SVM implementation was done in Python (https://www.python.org [as of 11 October 2021]) using the machine learning library scikit-learn^66^ (https://scikit-learn.org [as of 11 October 2021]). We used the *svm*.*LinearSVC*() function in its default configuration, i.e., with L2-norm regularisation and the squared Hinge loss function. For SVM training, we applied the sequential minimal optimisation (SMO) algorithm^67^. The kernel complexity C—a parameter regulating the trade-off between training error and generalisation ability—was optimised $\in \{10,1,10^{-1},10^{-2},10^{-3},10^{-4}\}$ to achieve the best performance on the test partition. Since SVMs are a two-class discrimination approach, for the three-class paradigm (d), binary one-vs-rest decisions were combined to generate the final decisions.

We tried out different classification system configurations with regard to common pre-processing steps. On the one hand, we compared audio normalisation prior to feature extraction by shifting each vocalisation’s signal mean to zero and setting its maximum amplitude to −3 dB^65,68,69^ vs using the unnormalised vocalisations. On the other hand, we tested three different feature normalisation options, namely infant-dependent normalisation vs global normalisation vs ‘feeding’ the classifier with unnormalised feature values. Feature normalisation was to rescale the values of each feature to the interval [0,1]. Applying the infant-dependent strategy, this was done in separate for the vocalisations of each participant. The global normalisation strategy implicated to include all vocalisations of the respective training partition. The identified minimum and maximum values were then applied to also normalise the feature values in the respective test partition. In addition to audio and feature normalisation, we tested training partition upsampling, i.e., systematic vocalisation reduplication in order to balance the number of vocalisations per participant in the training partition vs using imbalanced training partitions. Upsampling was done through integer multiplication of the vocalisations of each underrepresented participant to best possibly match the number of vocalisations of the participant with the highest number of vocalisations within a respective training partition.

Following the long lasting standard in the field of computational paralinguistics^46,51^, we selected the (class-)unweighted average recall (UAR) as the primary performance measure for classification evaluation. The UAR is calculated as the average of the class-specific recall values and represents the more adequate performance measure for class-imbalanced datasets as compared to the weighted average recall (accuracy). After each iteration of the cross-validation procedure, the vocalisation-wise decisions of the classifier were stored. Finally, the decisions of all iterations were combined and the UAR was calculated globally for all vocalisations of the dataset.

We evaluated two classification scenarios—both relevant for potential real-world applications (see “Discussion”): In the first scenario, the vocalisation-wise classification performance was evaluated. Thereby, we investigated if a participant’s developmental outcome can be detected from a single vocalisation. In the second scenario, we evaluated the classification performance on an infant basis, i.e., we generated an overall decision for each participant. This was done on the basis of the single decisions on all of his or her vocalisations by applying majority voting. According to this, a participant and, finally, all vocalisations of the participant were assigned to the class to which the most of his or her vocalisations were assigned. In the special case that no majority could be determined due to exactly the same number of decisions for different classes, we prioritised classes according to the arbitrarily defined order TD first—FXS second—RTT third. By evaluating the infant-wise scenario, we tried to find out if a participant’s developmental outcome can be detected from the sum of all of his or her vocalisations that are available.

#### Feature analysis

Introduced by Cortes and Vapnik in 1995^70^, SVMs represent classification units that make their class decisions based on a learnt hyperplane with maximal margin between the training data of the respective classes in the feature space. In linear SVM models, the coefficients of this hyperplane are set as weights, whose magnitude indicates the relevance of each feature in the decision function—the larger the magnitude of a feature weight, the more important the role the respective feature plays^71^. Accordingly, the sorting of features by the magnitude of feature weights has been described as a practical feature ranking procedure in linear SVM models^71,72^.

Following this procedure, we exported the feature weights of our generated classification models. Next, the 88 features were ranked according to the decreasing absolute values of their weights. For the two-class paradigms (a)–(c), the final feature ranking was based on the ascending mean of feature ranks obtained for the single iterations of the cross-validation procedure. As the model of the three-class paradigm (d) actually consists of three binary one-vs-rest sub-models, we calculated the mean of feature ranks obtained for the sub-models within each iteration of the cross-validation procedure first. Then, in a second step, we calculated the mean of the iteration-wise mean values and sorted the features accordingly in ascending order to create the final ranking. In case of exactly the same mean values for different features in any paradigm, ranking was arbitrarily defined to follow the internal feature index of the eGeMAPS in ascending order^63^.

## Results

We give the results for automatic vocalisation classification in Table 2. For each of the four investigated paradigms (a)–(d) (see “Analysis”), classification performance is reported for the system configuration, i.e., for the tested options on audio normalisation, feature normalisation, and training partition upsampling, that led to the highest complexity parameter C-dependent mean UAR of vocalisation-wise and infant-wise classification, respectively. For paradigms (a)–(c), the presented system configuration led to the respective highest UAR value in both the vocalisation-wise and the infant-wise scenario.

**Table 2:**
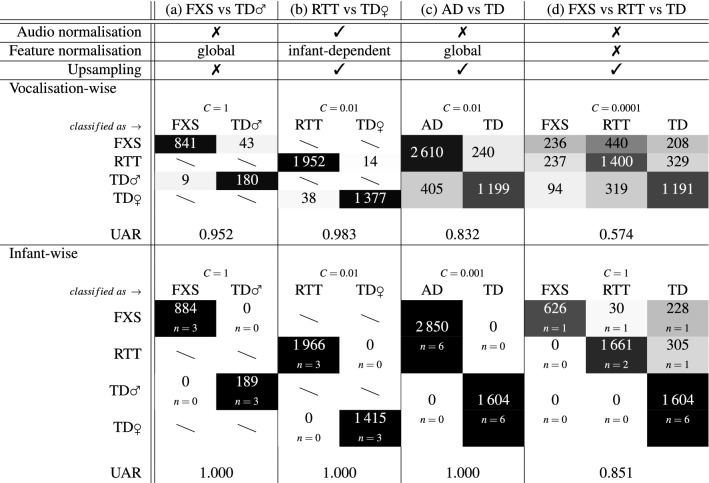
Paradigm-wise (a)–(d) best system configuration regarding audio normalisation, feature normalisation, and training partition upsampling, as well as respective classification results in form of class-specific numbers of (in)correctly assigned vocalisations (confusion matrices) and unweighted average recall (UAR) for both vocalisation-wise and infant-wise evaluation scenario.

UAR values are rounded to three decimal places. AD, atypical development; C, support vector machine kernel complexity parameter; FXS, fragile X syndrome; n, number of participants; RTT, Rett syndrome; TD, typical development; ♀, female;  ♂, male; colour code for confusion matrices: greyscale proportional to the class-specific number of vocalisations between white = 0 % of class-specific vocalisations and black = 100 % of class-specific vocalisations.

In all four paradigms, the achieved vocalisation-wise and infant-wise classification performance clearly exceeded chance level, i.e., a UAR of 50% for the two-class paradigms (a)–(c) and a UAR of $33.\dot{3}\%$ for the three-class paradigm (d).

In the vocalisation-wise evaluation scenario, the classification tasks FXS vs TD♂ and RTT vs TD♀ were solved at a UAR of 95.2% and 98.3%, respectively. For the task AD vs TD, a UAR of 83.2% was achieved at a higher proportion of misclassified vocalisations of TD participants than of AD participants. The three-class task FXS vs RTT vs TD resulted in a UAR of 57.4% with the highest proportion of misclassifications among vocalisations of participants with FXS. About the half of FXS-related vocalisations were assigned to the class RTT and almost 25% to the TD class.

In the infant-wise evaluation scenario, the two-class paradigms (a)–(c) were solved at a UAR of 100% meaning that all participants were correctly classified, respectively. In the three-class paradigm (d), 9 of 12 participants were correctly assigned to their respective classes, reflected by a UAR of 85.1%. One of the three participants with FXS—the one with the lowest number of vocalisations—was misclassified as a participant with RTT, another participant with FXS—the one with the second lowest number of vocalisations—as a TD participant. The participant with FXS with the highest number of vocalisations was correctly assigned to the FXS class. One of the three participants with RTT was incorrectly assigned to the TD class.

The achieved UAR of 100% in the infant-wise evaluation scenarios of paradigms (a)–(c) implicates that also all single iterations of the cross-validation procedure were solved at a UAR of 100 %, respectively. The models for the vocalisation-wise scenarios of paradigms (a), (b), and (d) performed at a UAR standard deviation of less than 4 percentage points across the single cross-validation iterations, respectively. The standard deviation of the iteration-wise UAR values for the vocalisation-wise scenario of paradigm (c) was 11.7 percentage points, for the infant-wise scenario of the three-class paradigm (d) 33.3 percentage points. Here, in one of the three cross-validation iterations, the model performed at chance level. This was the iteration, in which the quadruple of participants with the respective second highest class-specific number of vocalisations was used as test partition.

Regarding the tested classification pre-processing steps audio normalisation, feature normalisation, and training partition upsampling, no unique configuration was found to be similarly beneficial for all investigated paradigms. Using the unnormalised audio input data and performing training partition upsampling were each selected for three, however, not for the same three, but for two same paradigms as the respective best option. The two paradigms for which using unnormalisaed audio input data and at the same time performing training partition upsampling led to the best performance, were the two paradigms (c) and (d) that had all vocalisations of the dataset included. Global feature normalisation led to the best classification performance in two of the four paradigms. Infant-dependent normalisation and no feature normalisation at all were selected in one paradigm each.

Feature analysis results are reported as linked to the above presented best classification model configurations. For each of the four investigated paradigms (a)–(d), we list the respective top ten acoustic features of relevance for automatic vocalisation-wise and infant-wise classification in Table 3. As for the paradigms (a) FXS vs TD♂ and (b) RTT vs TD♀ the respective optimal SVM complexity parameter C was the same for both the vocalisation-wise and the infant-wise evaluation scenario, also the ranked features were the same for both scenarios.

**Table 3:**
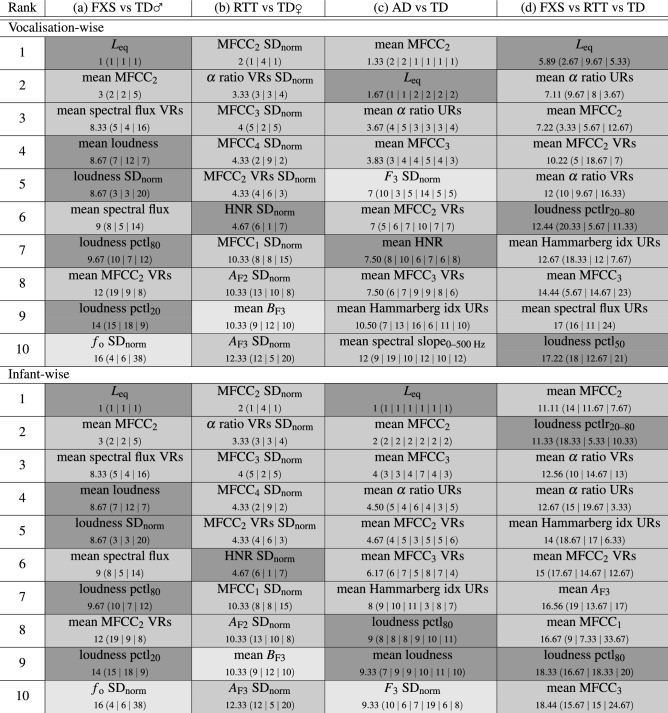
Paradigm-wise (a)–(d) top ten acoustic features according to the ascending mean (given below the respective feature name outside the brackets) of cross-validation iteration-wise (mean of sub-models) feature ranks (given below the respective feature name within the brackets) for both vocalisation-wise and infant-wise evaluation scenario.

Mean ranks are rounded to two decimal places. A, amplitude; AD, atypical development/atypically developing; B, bandwidth; $\text{f}_{o}$, fundamental frequency; $\text{F}_{2,3}$, second and third vocal formant; FXS, fragile X syndrome; HNR, harmonics-to-noise ratio; idx, index; $\text{L}_{eq}$, equivalent sound level; ${\text{MFCC}}_{1,2,3,4}$, first, second, third, and fourth Mel-frequency cepstral coefficient; pctl, percentile; pctlr, percentile range; RTT, Rett syndrome; $\text{SD}_{\text {norm}}$, standard deviation normalised by the arithmetic mean; TD, typical development; URs, unvoiced regions; VRs, voiced regions; ♀, female; ♂, male; colour code for features: light grey = frequency-related feature, middle grey = spectral/cepstral feature, dark grey = energy/amplitude-related feature.

Most of the top ten features for paradigm (a) FXS vs TD♂ were energy/amplitude-related features, such as the equivalent sound level or features based on the first-level descriptor loudness. Most of the top features across all other paradigms (b)–(d) were spectral/cepstral features, such as features based on MFCCs, the $\alpha$ ratio, the spectral flux, or formant amplitudes. One of the top ten features for each of the two-class paradigms (a)–(c) was frequency-related, namely based on the first-level descriptor $\textit{f}_{o}$, formant frequency, or formant bandwidth. None of the six temporal features of the eGeMAPS made it into the top ten features for any paradigm and scenario.

Most of the top ten features of paradigms (a), (c), and (d) were generated by applying the arithmetic mean to the trajectory of a first-level descriptor, whereas for nine of the top ten features for paradigm (b) RTT vs TD♀, the standard deviation normalised by the arithmetic mean was used as statistical functional for second-level feature processing.

Features based on the second Mel-frequency cepstral coefficient (MFCC_2_) turned out highly relevant across all paradigms. For each scenario, MFCC_2_-based features ranked among the top three features with a good consistency between the ranks obtained for the single iterations of the cross-validation procedure. Similarly, the equivalent sound level played an important role in the automatic vocalisation-wise and infant-wise classification models of paradigms (a) FXS vs TD♂ and (c) AD vs TD, as well as for the vocalisation-wise classification model of paradigm (d) FXS vs RTT vs TD.

**Figure 1:**
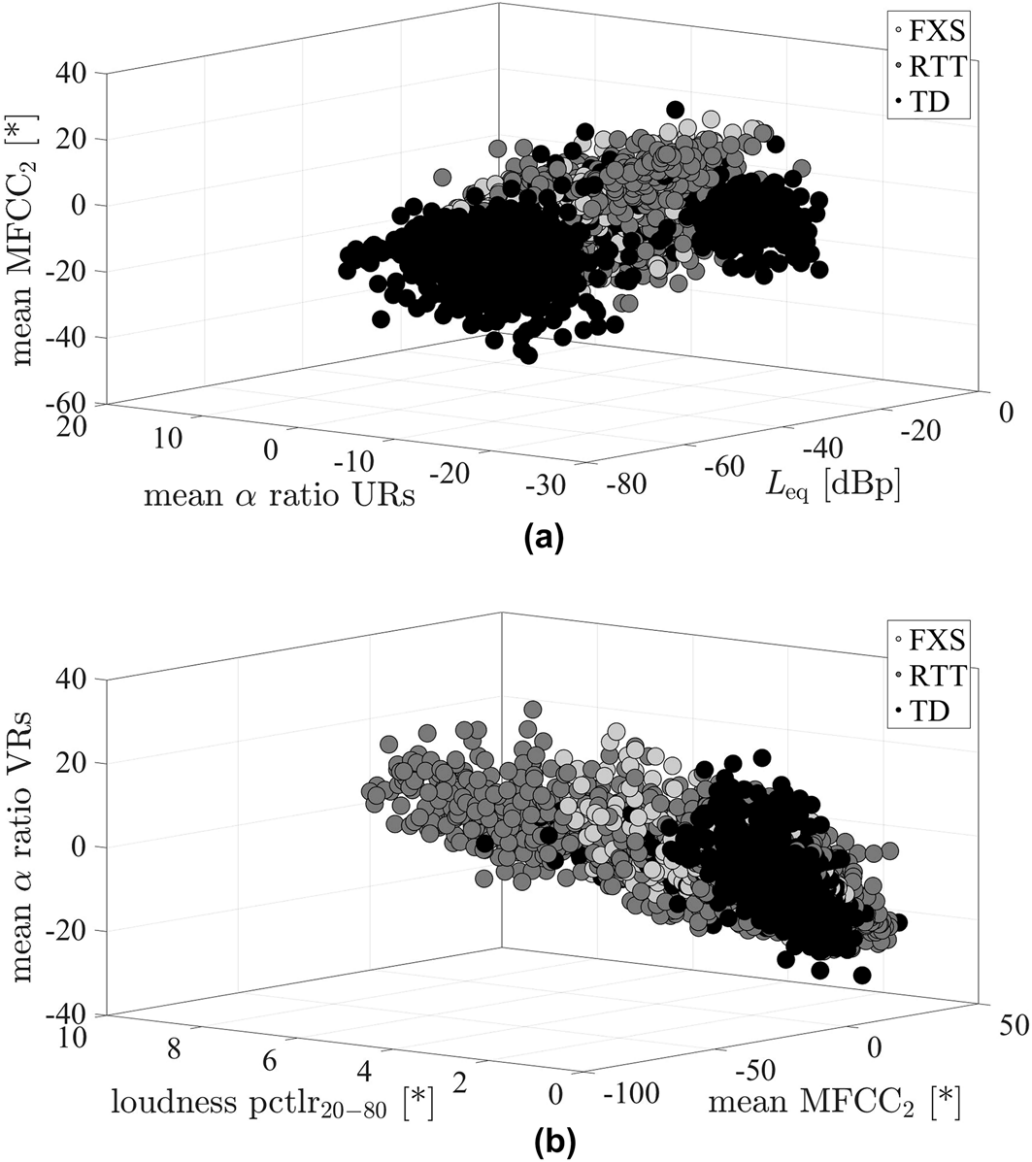
Vocalisation distributions for the paradigm FXS vs RTT vs TD within the space of the three best ranked acoustic features for (**a**) the vocalisation-wise and (**b**) the infant-wise evaluation scenario, respectively (see Table 3), in best system configuration, i.e., without audio and feature normalisation (see Table 2). dBp power converted to decibel with $10 \times log_{10}(x)$, FXS, fragile X syndrome, $\text{L}_{eq}$, equivalent sound level, ${\text{MFCC}}_{2}$, second Mel-frequency cepstral coefficient, pctlr, percentile range, RTT, Rett syndrome; TD, typical development; URs, unvoiced regions; VRs, voiced regions; *Real measurement unit not existent as feature values refer to the amplitude of the digital audio signal.

Figure 1 exemplarily illustrates the vocalisation distributions for the three-class paradigm (d) FXS vs RTT vs TD within the space of the respective three top ranked acoustic features for the vocalisation-wise and the infant-wise evaluation scenario. Although only based on three features, it already reveals a rough clustering according to different classes. In both the vocalisation distributions for the vocalisation-wise and the infant-wise scenario, the TD class and the RTT class can be reasonably well discriminated. Conforming to the situation of misclassifications (see Table 2), in the vocalisation-wise evaluation scenario vocalisations of individuals with FXS are spaciously overlayed with the vocalisations of individuals with RTT. In the infant-wise evaluation scenario, the FXS cluster rather overlaps with the TD cluster.

## Discussion

In this study, we aimed to investigate whether an automatic early verbal behaviour-based differentiation of FXS and/or RTT vs TD is feasible. Therefore, we evaluated four classification paradigms in two scenarios and tried out different classification system configurations on the basis of a compact set of retrospectively collected vocalisation data. Our classification results—especially, more than 95 % UAR for the two-class paradigms (a) FXS vs TD♂ and (b) RTT vs TD♀ in the vocalisation-wise evaluation scenario as well as 100 % UAR for all three two-class paradigms in the infant-wise evaluation scenario—not only demonstrate basic feasibility, but point to the high potential of the approach for future practical application in paediatric healthcare. Even the three-class paradigm yielded promising results that motivate an extension for further classes of late detected developmental disorders to be modelled and discriminated. However, for a paradigm on several disorders and TD it might be more beneficial to discriminate between TD and AD by means of anomaly detection ^73^ in a first step, and then to further discriminate between the different developmental disorders in a multi-class paradigm in a second step.

The present study was the very first attempt to combine early vocalisation data of individuals with different late detected genetic disorders within one classification model, the first to apply intelligent audio analysis methodology to early vocalisation data of individuals with FXS, and—following our 2016 study^55^—the second attempt to automatically identify early vocalisations of individuals with RTT. In our former study^55^, we investigated the vocalisation-wise classification paradigm RTT vs TD♀ by also training and evaluating linear kernel SVMs on the basis of a LOSpO cross-validation scheme. Both, our 2016 study and the RTT-associated paradigms of the present study built upon the audio-video material of the same participants with RTT and female TD controls, except from the material of one participant pair of a female TD control individual and an individual with the relatively milder preserved speech variant (PSV) of RTT^40^ that was not included in the present study for better diagnosis homogeneity across the dataset. In our 2016 study, we extracted the 6373 official baseline features of the annual (Interspeech) Computational Paralinguistics Challenges since 2013 (ComParE set)^49^, we tried out infant-dependent feature normalisation and used an upsampling technique to minimise class imbalances in the training sets. In the present study, both infant-dependent feature normalisation and training partition upsampling turned out to be the best option to discriminate between the classes RTT and TD♀. In our 2016 study^55^, we achieved a mean UAR over the single cross-validation iterations of 76.5 % at a standard deviation of 23.4 % compared to a mean UAR of 97.5 % at 3.5 % standard deviation for the vocalisation-wise scenario of paradigm (b) in the present study (referring to the reported global UAR of 98.3 %). The clearly better classification performance in combination with a much higher robustness across the cross-validation iterations in the present study might come from (i) only having used three instead of four participant pairs by having excluded the pair with the individual with the PSV of RTT, (ii) the exclusion of all positive- and negative-mood vocalisations from the present study, (iii) having used a more compact and specialised feature set, and/or (iv) audio input normalisation that was not tested in our 2016 study, but turned out to be the better option for the paradigm RTT vs TD♀ in the present study than using unnormalised audio input data.

A key challenge in automatically discriminating early verbal behaviour of TD individuals and individuals with a late detected developmental disorder, especially with RTT, is the proportion of vocalisations of affected individuals that appear inconspicuous—at least for a human listener^39,42,43^. By tolerating almost every second of an affected individual’s vocalisations to be classified as typical, infant-wise classification through majority voting on single vocalisation decisions might therefore be a good evaluation model in step with actual practice—given a setting in which a sufficient number of vocalisations of an individual can be recorded. Thereby, also TD individuals can ‘afford’ to produce some vocalisations classified as atypical before they are assumed to be atypically developing. This is an essential point with regard to real-world applications as informing parents about the potential AD of their children represents a very sensitive issue. Instead of applying majority voting to come to a final infant-wise decision, the decision threshold could also be specifically adjusted/optimised for each modelled disorder. This approach turned out beneficial for the automatic verbal behaviour-based classification paradigm ASD vs TD^74^.

For a deeper understanding of the acoustic models behind the trained classifiers, we itemised acoustic vocalisation characteristics to appear maximum relevant for class discrimination. In doing so, we utilised the computer to unravel what the human listener might not be able to hear. The gained information does not only give us the chance to optimise existing acoustic feature sets for specific paradigms/scenarios or to design new efficient task-specific feature sets, but to derive knowledge of voice-physiological processes characterising the early verbal development of individuals with FXS or RTT, or potentially any other late detected developmental disorder. However, caution is needed when interpreting results for the three-class paradigm (d) FXS vs RTT vs TD, as the underlying SVM weights refer to non-normalised features. Moreover, it has to be kept in mind that several features of the eGeMAPS are correlated with each other as they, for instance, rely on the same first-level descriptor (e.g., different statistical functionals calculated for the $\textit{f}_{o}$ contour).

Most identified top features turned out robust across the single iterations of the cross-validation procedure. This argues for features that are in fact associated with the individual’s medical condition and not, for example, with potential acoustic (background) irregularities in single partitions. When interpreting the robustness of top features across the different paradigms, one should not forget that one and the same data were each (re)used in three of the four examined paradigms, such as the vocalisations of individuals with FXS in paradigms (a) FXS vs TD♂, (c) AD vs TD, and (d) FXS vs RTT vs TD. The paradigms (a) and (b) were the only paradigms for which the used data did not overlap and none of the identified ten top features were the same. This suggests that early verbal peculiarities of individuals with FXS and individuals with RTT acoustically manifest in different ways as compared to typical early verbal behaviour of gender-matched controls. However, for a more reliable picture, also potential acoustic differences between the early verbal behaviour of male and female TD individuals^75^ would have to be considered. Here, it seems as if RTT-associated early verbal behaviour primarily differs from early verbal behaviour of female TD individuals by means of the variation of spectral/cepstral first-level descriptors within a vocalisation. This becomes manifest (i) in the standard deviation normalised by the arithmetic mean to be in nine of the top ten features the used statistical functional for second-level feature processing and (ii) in eight of the top ten features to rely on spectral/cepstral first-level descriptors. However, the proportion of spectral/cepstral features being among the top ten features has to be interpreted with caution as almost half of the 88 eGeMAPS features are spectral/cepstral features. In contrast to the paradigm RTT vs TD♀, the model of discriminating individuals with FXS against male TD individuals essentially built upon energy/amplitude-based first-level descriptors and the arithmetic mean as the most frequently used statistical functional among the top ten features.

### Limitations

In this study, we provided the technical underpinnings for a novel approach to (i) automatically classify FXS and/or RTT vs TD based on early verbal behaviour and (ii) to gain new insights into early syndrome-related vocalisation characteristics. However, even though our findings indicate that this approach is worth to be further followed, they have to be interpreted carefully and can hardly be generalised. This is primarily due to the very small dataset in terms of number of vocalisations and the definitively more problematical number of age, gender-, and family language-controlled participants per class available for experimentation. However, it has to be kept in mind that the acquisition of prodromal vocalisation data of individuals with a rare late detected genetic disorder represents a challenging endeavour. Anyway, to guarantee that each classification model was trained on data of at least two participants per (sub-)class (FXS, TD♂, RTT, TD♀), we were forced to abstain from a separate development partition and had to directly optimise the classifier on the test partition. Consequently, model evaluation cannot be regarded as independent from model training. A further shortcoming of our study is the highly imbalanced number of participant-wise vocalisations that can be essentially explained by the varying duration of available audio-video material across participants. By testing the system configuration option of training partition upsampling, we tried to counter single participants to be underrepresented for class modelling in terms of data quantity. Another issue coming from the availability of audio-video material is the inhomogeneous distribution of vocalisations over age across participants in combination with a relatively broad investigation age window of 6 months. It must be considered that verbal development in the second half year of life allows for a range of acoustically and/or sequentially differing age-adequate vocalisation types, e.g., single vowels vs canonical babbling^7^, while the numbers of available vocalisations per type across participants of our study might be very different. Finally, the used audio-video material itself to consist of non-standardised home video clips potentially introduces bias due to variations in recording device/format-dependent raw audio quality, background noise, microphone level, recording setting, recording location, etc. Moreover, it has to be kept in mind that the recordings were originally not made with the parents’ intention to collect data for later scientific analyses, but typically to create a memory of family routines and special moments of their children’s infancy. Thus, the material underlies a bias of parental scene pre-selection. As most parents might tend to turn off the camera when their children start to behave somehow peculiarly, it can be assumed that apparently atypical verbal behaviour—especially in the included individuals with FXS and RTT—might be unnaturally underrepresented in the dataset^76,77^. Nevertheless, the retrospective analysis of home video material provides the unique chance to study early development in a natural setting and currently represents the best available approach to objectively investigate prodromal behavioural phenomena in rare late detected developmental disorders such as FXS or RTT^56,77–79^.

## Perspectives

Using the example of FXS and RTT, our study can be regarded as a technical contribution to a future earlier detection of adverse development and the identification of atypical vocalisation characteristics. We could demonstrate that, even if the studied individuals with FXS and RTT had not yet shown clinical signs in their second half year of life, the machine ‘hears’ that they had already vocalised differently from TD individuals. One of the most important next steps will be to collect large quantities of further vocalisation data. Apart from an increasing reliability and generalisability of the results, a significantly larger dataset will offer the opportunity (i) to train separate models for small age windows, e.g., of single months, and/or for specific vocalisation types, such as for canonical babbling^6,7^, (ii) to evaluate classification models entirely independently from the training process by using separate development partitions, and (iii) to apply more sophisticated, state-of-the-art machine learning methodology, such as deep learning approaches^80^, which have recently found their way into the field of medical research and have proven well for various disease detection/classification tasks^81,82^. The influence of family language and culture on the presented approach should be also focused on in future work. We assume that family language- and culture-conditioned differences in the distribution of sounds and sound sequences produced in the second half year of life, might also cause differences in the acoustic manifestation of atypical verbal behaviour and, therefore, differences in the machine learning model’s weighting of acoustic features. Thus, either a universal model comprising various languages and cultures, or individual models for different languages and cultures should be tested on the basis of appropriate data. Finally, an extension of the present study for further late detected developmental disorders would be of scientific interest and of high value for real-world applications.

Irrespective of data quantity and the concrete application scenario, it has to be considered that intelligent audio analysis underlies probabilistic modelling and, therefore, allows for misclassification. For a more reliable performance, we suggest a multimodal approach that exploits verbal input data in combination with data from other developmental domains^58^. In any case, the presented work provides the foundation for a future assistive tool that raises suspicion of AD or specific developmental disorders to potentially initiate a diagnostic cascade or even predicts individual outcomes.
